# Ursolic Acid-Based Derivatives as Potential Anti-Cancer Agents: An Update

**DOI:** 10.3390/ijms21165920

**Published:** 2020-08-18

**Authors:** Vuyolwethu Khwaza, Opeoluwa O. Oyedeji, Blessing A. Aderibigbe

**Affiliations:** Department of Chemistry, University of Fort Hare, Alice Campus, Alice 5700, Eastern Cape, South Africa; 201209227@ufh.ac.za (V.K.); ooyedeji@ufh.ac.za (O.O.O.)

**Keywords:** ursolic acid, derivatives/analogs, structural modification, anti-cancer activity

## Abstract

Ursolic acid is a pharmacologically active pentacyclic triterpenoid derived from medicinal plants, fruit, and vegetables. The pharmacological activities of ursolic acid have been extensively studied over the past few years and various reports have revealed that ursolic acid has multiple biological activities, which include anti-inflammatory, antioxidant, anti-cancer, etc. In terms of cancer treatment, ursolic acid interacts with a number of molecular targets that play an essential role in many cell signaling pathways. It suppresses transformation, inhibits proliferation, and induces apoptosis of tumor cells. Although ursolic acid has many benefits, its therapeutic applications in clinical medicine are limited by its poor bioavailability and absorption. To overcome such disadvantages, researchers around the globe have designed and developed synthetic ursolic acid derivatives with enhanced therapeutic effects by structurally modifying the parent skeleton of ursolic acid. These structurally modified compounds display enhanced therapeutic effects when compared to ursolic acid. This present review summarizes various synthesized derivatives of ursolic acid with anti-cancer activity which were reported from 2015 to date.

## 1. Introduction

At present, cancer remains a significant health problem and is the second leading cause of death worldwide. The Global Cancer Incidence, Mortality and Prevalence (GLOBOCAN) recently reported that in 2018 about 18.1 million people were diagnosed with cancer and more than 9.6 million deaths were reported due to cancer [1]. Made et al. indicated that by 2025, the number of people living with cancer will increase [2]. In general terms, cancer is defined as a tumor resulting from the abnormal proliferation of cells that can spread to various organs of the body. Most of the currently used treatment includes the combination of chemotherapeutic agents, surgery, radiotherapy, or hormone therapy. Despite the above treatment strategies, there are still many limitations associated with cancer treatment such as multi-drug resistance, unselective targeting of the cancer cells, drug toxicity, etc. [3].

The shortage of suitable cancer chemopreventive procedures suitable for improving the therapeutic outcomes during an anticancer treatment has motivated researchers around the world to test the anticancer effect of biomolecules obtained from natural sources. Natural products are one of the main sources of pharmacologically active compounds, and they are potentially useful for the development of drugs. Generally, natural products are more environmentally friendly for frequent use when compared to synthesized drugs [4]. Extensive research has been performed to study the therapeutic effect of drugs derived from plants against a number of cancers, and the outstanding results indicate that most natural active compounds have potent anticancer effects [5]. Natural compounds can inhibit the formation and development of cancer by specifically interacting with multiple cell-signaling pathways [6]. These properties make it possible to affect multiple cancer hallmarks [7]. As a result, about 74% of FDA-approved drugs are either natural products or natural product-derived [8]. Active chemical compounds originally isolated from natural products contain some dominant structures which can be modified to form new effective drugs [9].

A class of natural products called triterpenoids is among the most important group of phytochemicals originally derived from plants, with approximately 20,000 chemical structures confirmed so far [10]. Triterpenoids comprise six isoprene units with a basic molecular formula (C_30_H_48_). In terms of biological perspectives, the large group of pentacyclic triterpenoids with basic molecular structures with five membered rings have attracted the attention of many researchers due to their notable broad spectrum of pharmacological activities including anticancer, anti-inflammatory, antioxidant, antiviral, antimicrobial, etc. [11,12].

Ursolic acid (UA) 3-(β-hydroxy-urs-12-en-28-oic acid) is a ubiquitous pentacyclic compound that possesses functional groups such as a carboxylic moiety at C28, β-hydroxy function at C3, and an alkene at C12-C13. UA was first identified in the 1920s from the epicuticular waxes of apples [13,14] and it has been isolated in recent years from many other plant organs. Some plant species containing UA as an active constituent are listed in Table 1.

**Figure 1:**
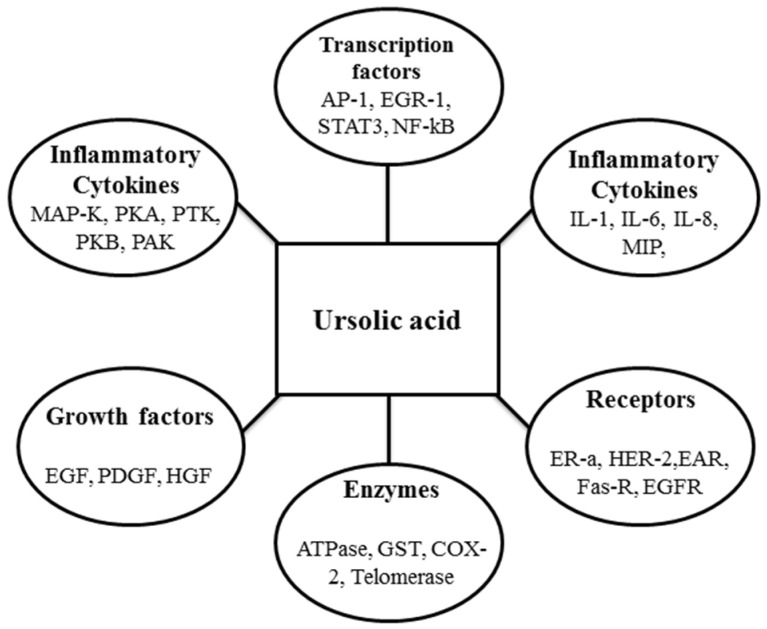
Multiple molecular targets modulated by UA.

UA possesses various interesting biological activities including anticancer, anti-inflammatory, antimicrobial, antidiabetic properties etc. [65,66,67,68]. Some reports have extensively explored the pharmacological properties of UA, as shown in Table 2. In terms of the anticancer properties, studies have demonstrated that UA can modulate the cellular transcription factor; growth factor receptors; cytokines inflammatory; and many other molecular targets which regulate the cell proliferation, metastasis, apoptosis, angiogenesis, and autophagy of cancer cell lines through different mechanisms and signaling pathways [69,70] The anticancer effects of UA have been reported for various types of cancers such as endometrial [71,72], pancreatic [73,74], lung [75,76,77], prostate [78,79,80], ovarian [81,82], bladder [83], gastric [84,85], and liver cancers [86]. Other UA molecular targets reported for the treatment of cancer include its effect on p53 pathways [87,88,89,90]; the canonical pathway (Wnt/β-catenin) [91,92]; Ras signaling [93]; and transcription pathways like nuclear factor kappa light chain enhancer of activated B cells (NF-Kb) [94], Tumor Necrosis Factor-Related Apoptosis-Inducing Ligand (TRAIL) [95], and the Signal transducer and activator of transcription 3 (STAT3) family of transcription factors [96,97,98]. Figure 1 below depicts the various molecular targets regulated by UA.

The biopharmaceutical classification system (BCS) classified UA as a class IV drug with limited pharmacological effect due to its low water solubility and difficulty in permeating some biological membranes. Drugs in this class not only have slow dissolution but also have limited gastrointestinal mucosa penetration, which results in their low oral bioavailability [99,100]. Due to the aforementioned reasons, most researchers have developed some new strategies to enhance the biopharmaceutical effect of UA via loading in nanoformulations or the structural modification of its structure. Chen et al. reported various derivatives of UA with potential anticancer activities until 2015 [101]. The main contents of the present review provide an update on reported UA derivatives with potential anticancer activities reported over the last five years (2015–2020).

## 2. An overview of Pharmacokinetics of UA and Its Derivatives

As mentioned earlier, UA is among the most studied triterpenoids and possesses a wide range of pharmacological activities, but its poor water solubility hinders its efficacy and potential as an appropriate therapeutic drug [137,138]. To overcome its poor water solubility, a number of structure–activity relationships (SARs) of the synthesized derivatives have been studied. The structural modification of UA is a suitable approach to enhance its pharmacokinetic profiles. UA and its derivatives have numerous pharmacological activities and most of them have been extensively screened and reviewed in recent published articles and reviews [101,138]. In terms of toxicity, UA and its derivatives are safe phytochemical compounds with low toxicity. In vitro studies have revealed the cytotoxic activity of UA and its derivatives on tumor cell lines and low cytotoxic effects against normal cell lines [139,140]. Additionally, the in vivo toxicity analysis of UA in animals showed no sign of toxicity in Kunming mice (0.2 mL/10 g) [141]. UA displayed a significant anticancer activity in animal models in vivo [142,143]. Several studies have reported the pharmacokinetics of UA and its derivatives in different animal models. Alzate and colleagues used Phoenix WinNonlin software to determine the pharmacokinetic profile of UA administered to Wistar rats at different doses and routes (i.e., 1 mg/kg intravenously and 20 and 50 mg/kg orally). The results indicated that the oral bioavailability of UA was significantly different between the two groups that were administered UA orally at doses of 20 mg/kg (2.8%) and 50 mg/kg (1.55%) [144].

Liao et al. developed and validated a rapid, sensitive, and accurate liquid chromatography–mass spectrometry (LC–MS) method for the determination of ursolic acid in rat plasma. In this procedure, rat plasma was acidified with acetic acid and then extracted with a mixture of hexane-dichloromethane-2-propanol (20:10:1, *v*/*v*/*v*). This LC-MS method was successfully used for pharmacokinetic studies after the oral administration of a Lu-Ying extract containing 80.32 mg/kg UA to the rats [145]. Furthermore, UA was established as an internal standard to determine the glycyrrhetic acid and gambogic acid in human plasma to determine their pharmacokinetics using sensitive liquid chromatography-electrospray ionization-mass spectrometry (LC–ESI-MS) [146,147]. However, a study showed that, after the administration of a UA solution, liposome-loaded UA, or chitosan-coated UA liposome (CS-UA-L) to mice models via the intra-gastric route, the content of UA was high in the liver, spleen, and kidney for the group administered the UA solution when compared to those administered with the UA liposome and CS-UA-L groups. However, a significantly greater amount of UA was accumulated at the tumors for the mice treated with CS-UA-L, which was 4.2- and 1.7-fold higher than those administered the UA solution and UA liposome groups, respectively. CS-UA-L significantly and selectively accumulated in the tumor tissues [148].

Interestingly, UA-medoxomil (NX-201), a UA prodrug (a derivative of UA with a structural modification of C-28 position) showed an improved bioavailability about 200 times better than UA in a rodent model. According to an in vivo test performed with a human pancreatic cancer (PANC-1) xenograft Severe Combined Immunodeficient (SCID) mouse model, the tumor growth rate decreased in a dose-dependent approach and a 100 mg/kg dose of NX-201 had an anticancer effect comparable to gemcitabine [149]. Despite the various reports on the pharmacokinetics of UA and its derivatives, there are currently few clinical trials. UA has been loaded into liposomes and there are few reports [150,151,152,153].

### Clinical Trials of UA

A few clinical trials have been conducted to assess the pharmacokinetics of various UA formulations using both healthy volunteers and patients with different types of cancers. UA is currently undergoing phase I trials to investigate its safety and adverse effects in patients.

Wang et al. investigated liposomal ursolic acid (LUA) pharmacokinetics, maximum tolerated dose (MTD), and dose-limiting toxicity (DLT) in healthy adult volunteers and patients with advanced solid tumors. A total of 63 subjects (i.e., 35 healthy volunteers, 24 adults, and 4 patients) received a single dose of LUA (11, 22, 37, 56, 74, 98, and 130 mg/m^2^) administered as a 4 h intravenous infusion. The clinical data indicated that LUA had low toxicity with a MTD of 98 mg/m^2^ [152]. Other studies demonstrated that incorporating UA into liposomes can increase the ceramide content of the human subject’s skin, with an increase in the hydroxyl-ceramides appearing after three treatments [153]. Zhu et al. also investigated the single and multiple-dose pharmacokinetics and the safety of UA nanoliposomes (UANL) in 24 healthy volunteers and 8 patients with advanced solid tumors. The 24 healthy volunteers were divided into three groups which received a single dose of 37, 74, and 98 mg/m^2^ of UANL. Eight patients received multiple doses of 74 mg/m^2^ of UANL daily for 14 days. Ultra-performance liquid chromatograph-tandem mass spectrometry was used to determine the UA plasma concentrations [150]. The UANL was found to be safe and showed apparent linear pharmacokinetics (PK) behavior for a dose level ranging from 37 mg/m^2^ to 98 mg/m^2^. The repeated UANL administration indicated no drug accumulation even with 14 days of continuous IV infusion. Patients with solid tumors and healthy volunteers tolerated the IV infusion of UANL very well [150]. These studies clearly suggest that UA has tremendous potential to be developed into a potent anticancer drug.

## 3. Chemistry of UA

The structure of a pentacyclic triterpenoid UA comprises C-30 isoprenoid; it has a low water solubility but is highly soluble in alcoholic NaOH and glacial acetic acid. It is a white crystalline solid with a melting point and molecular weight of 284 °C and 456.70,032 g/mol, respectively. Its maximum UV absorption wavelength is ~450 nm [154].

### 3.1. UA Derivatives as an Anticancer Agent

**Figure 2:**
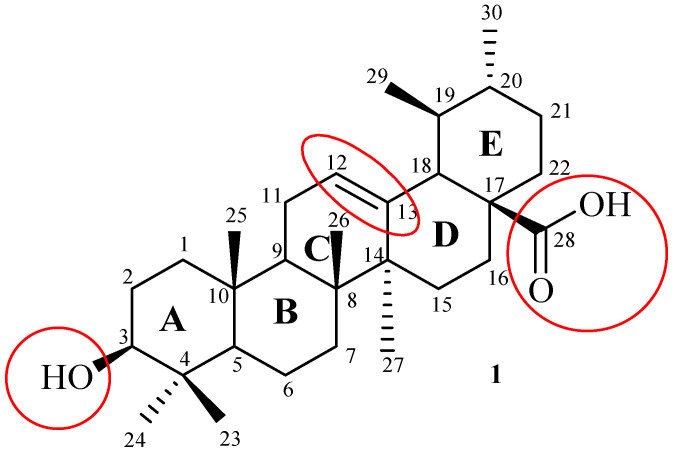
Structure of UA indicating the major active sites.

Many researchers have studied various strategies to modify the molecular structure of UA and simultaneously enhance its therapeutic effect. The structure of UA has been broken down into functional groups or pharmacophores to classify the major active sites for structural modification (Compound **1**, Figure 2). These sites are broadly categorized as the carboxylic moiety (C-28), β-hydroxy function (C-3), and an alkene at C12–C13. The sites have been extensively studied for their potential anti-cancer activity. Some derivatives that are not identified in any of the aforementioned three pharmacophores are separately categorized into miscellaneous groups. The following reports further detailed the literature previously reported under these active sites and their successfully enhanced anticancer activity.

#### 3.1.1. Modification of the Carboxylic Moiety (C-28)

**Scheme 1:**
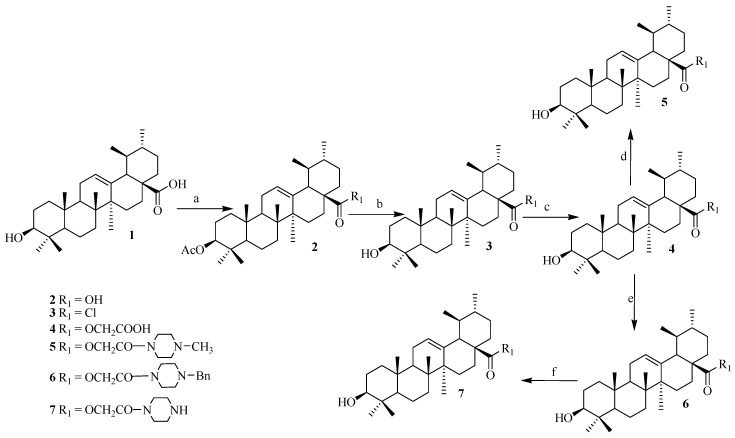
Reagents and conditions: (**a**) Ac_2_O, DMAP, THF, rt; (**b**) (COCI)_2_, CH_2_Cl_2_. Rt; (**c**) HOCH_2_ COOH, TEA, rt; (**d**) N-methylpiperazine, EDCI, DMAP, CH_2_Cl_2_, 0 °C to rt; (**e**) Benzylpiperazine, EDCI, DMAP, CH_2_Cl_2_, 0 °C to rt; (**f**) 10% Pd/C, H_2_ anhydrous ethanol, rt.

**Scheme 2:**
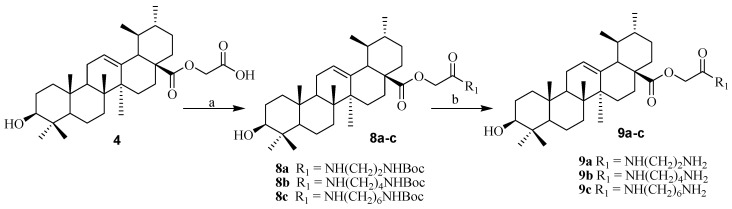
Reagents and conditions: (**a**) N-Boc-Diamine, EDCl, DMAP, CH_2_Cl_2_, 0 °C to rt; (**b**) TFA, CH_2_Cl_2_, 0 °C to rt.

Tian et al. synthesized a series of UA derivatives bearing diamine moieties at C-28 and investigated their antiproliferative potential against three human cancer cell lines (MCF-7, HeLa, and A549). This study was performed as a comparative analysis against the conventional antitumor drug, gefitinib. The derivatives were prepared by first protecting the C3-OH via acetylation, followed by esterification with 2-hydroxyacetic acid at the C-28 position and amidation reaction with amines including piperazine, *N*-methylpiperazine and alkane-1, 6-diamines and alkane-1, 4-diamines and alkane-1, and 2-diamines, as shown in Scheme 1 and Scheme 2. The half maximal inhibitory concentration (IC_50_) values for most of these derivatives were significantly higher when compared to gefitinib. The study revealed that derivatives containing primary amine moieties were more effective when compared to those with secondary or tertiary amine moieties. The antiproliferative activities all of the secondary amines were more active than those of the tertiary amine compounds. Compound **9a** (Scheme 2, Table 3) showed a more potent antiproliferative activity than other derivatives [155].

**Scheme 3:**
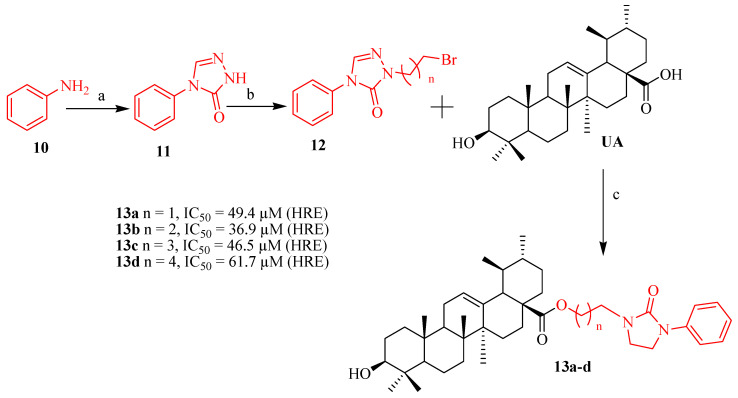
Synthetic route for compounds **10**–**13**. Reagents and conditions: (**a**) NH_2_NHCOOCH_3_, CH(OC_2_H_5_)_3_; Ethanol(EtOH), MeONa, reflux, 48 h; (**b**) Br(CH_2_)nBr; Potassium carbonate (K_2_CO_3_), KI; (**c**) K_2_CO_3_, KI; (CH_3_)_2_CO, reflux, 10 h.

A series of new derivatives of UA bearing oxadiazole, piperazine, and triazolone moieties was designed by Chi et al. and they were evaluated for their anticancer activity as Hypoxia-inducible factor-1α (HIF-1α) inhibitors. The in vitro results revealed that these derivatives had a significantly enhanced antitumor activity by inhibiting the expression of HIF-1α. Compound **13b** (Table 3) demonstrated the best potent inhibitory effect against HIF-1α activity but was not cytotoxic to cancer cells. These results indicated that the simple esterification of the UA carboxyl moiety can result in a significantly enhanced inhibitory effect on HIF-1α activity and decreased toxicity. The mechanism of action suggested that **13b** can also suppress cell proliferation and block cell cycle progression in the G1 phase (Scheme 3) [156].

Liu et al. synthesized a number of novel UA derivatives and evaluated their antitumor activities against two human cancer cell lines—gastric cancer cells (MGC-803) and breast cancer cells (Bcap-37) using an MTT assay in vitro [157]. These derivatives were obtained by reacting UA at C-28 with 1,2-dibromoethane, 1,3-dibromoethane, 1,4-dibromoethane, and butyl bromide in a solution of DMF and K_2_CO_3_, followed by reaction with corresponding amines. Most of the derivatives exhibited moderate to high inhibitory activities when compared to UA. The results illustrated that compound **14** (Table 3) induced cell apoptosis in MGC-803 cells.

#### 3.1.2. Modification of both β-hydroxy (C-3) and Carboxylic Moiety (C-28)

Hua et al. synthesized a series of new UA derivatives by incorporating piperazine and thiourea at the C28 position and evaluated their in vitro cytotoxicity against selected cancer cell lines—namely HCT-116, T24, MGC-803, HepG2, and A549 cells and a normal cell line, HL-7702, using a 3-(4,5-dimethylthiazol-2-yl)-2,5-diphenyltetrazolium bromidefor (MTT) assay. The in vitro antiproliferative results suggested that most of the derivatives exhibited a potent inhibitory effect. However, one compound displayed an excellent in vitro cytotoxicity on HepG2 cells with an IC_50_ value of 5.40 µM and significantly induced apoptosis in the HeG2 cell line (compound **15**, Table 4) [139]. Wiemann et al. designed a class of ursolic and oleanolic acid-derived hydroxamates in an attempt to investigate their cytotoxicity, using sulforhodamine B (SRB) assays against several human cancer cell lines (518A2, A2780, A549, FaDu, HT29, MCF-7, and NIH 3T3). The ursolic acid-based derivatives containing at least an OH and NH moiety in the hydroxamate part displayed good cytotoxicity and were significantly less selective to the cancer cells when compared to the oleanolic acid-based compounds. The results showed that compound **16** (Table 4) was the most potent compound, with IC_50_ values ranging from 2.5 to 6.4 µM [158].

Nedopekina et al. synthesized conjugates of triterpenoids UA and betulinic acid with the triphenylphosphonium (TPP^+^) group; evaluated their cytotoxic activity against two human cancer cell lines; and also studied their ability to induce programmed cancer cell death by employing markers of apoptosis, including the activation of caspase-3, permeabilization of the outer mitochondrial membrane, PARP-1 cleavage, the release of cytochrome *c,* and the inhibition of the mitochondrial respiratory chain. Two of the conjugates derived from ursolic acid and betulinic acid indicated a good range of IC_50_ values. Compound **17** (Table 4) derived from UA was obtained by the alkylation of the carboxyl group, C-28 of UA, with triethylene glycol dibromide in dimethylformamide (DMF) using K_2_CO_3_ at 50 °C for 3 h [159]. Jiang et al. synthesized a series of UA derivatives as inhibitors of Nuclear factor kappa B (NF-κB) by introducing long-chain amide moieties at the C-28 position, and the β-hydroxy group C-3 was protected by an acetyl group. Their in vitro anticancer properties and Tumor necrosis factor alpha (TNF-α-induced) NF-κB activation were evaluated against four human cancer cell lines, such as human ovarian cancer cells (SKOV3), lung cancer cells (A549), liver cancer cells (HepG2), and bladder cancer cells (T24). Several compounds exhibited considerable anticancer effects against different cancer cell lines. Compound **18** (Table 4) demonstrated the highest potency by inhibiting the growth of SKOV3, A549, HepG2, and T24 cells with IC_50_ values of 8.95, 5.22, 6.82, and 6.01 μM, respectively. The IC_50_ values were five-fold to eight-fold lower than the parent UA, and the results showed that compounds with longer diamide side chains showed relatively enhanced activity compared to compounds with shorter diamide side chains. A related mechanism study indicated that compound **18** caused cell cycle arrest at the G1 phase and triggered apoptosis in A549 cells by blocking the NF-κB signaling pathway [94].

Zhang et al. designed and synthesized UA-based tetrazole derivatives and studied their potential antitumor effects as HIF-1α transcriptional inhibitors. Compound **19** was the most potent with (IC_50_ = 0.8 µM) (Table 4). This compound was prepared by mixing the corresponding anhydrides with UA in a solution of trimethylamine and 4-dimethylaminopyridine (DMAP) in ice and stirred overnight at room temperature; the intermediate compound was reacted with SOCl_2_ in refluxing dichloromethane (DCM). The results suggest that introducing tetrazole at C-28 of UA increased the HIF-1α inhibitory effect; additionally, the introduction of large groups at C-3 is disadvantageous for the synthesis of effective HIF-1α inhibitors [160]. Kahnt et al. studied the ethylenediamine-spaced carboxamides of UA and betulinic acid and further analyzed their cytotoxicity effects against human tumor cells (8505C, A2780, MCF-7, HT29, NIH 3T3, and 518A2). The UA derivatives that demonstrated good in vitro cytotoxicity effects were compounds **20**, **21**, and **22**) [161].

Wang et al. evaluated the antiproliferative activity of UA derivatives containing thiazole, triazole, tetrazolepiperazine, or homopiperazine moiety against two cancer cells, Hela and MKN45. Compound **23** (Table 4) displayed a superior antiproliferative activity on both cell lines and induced apoptosis via the intrinsic mitochondrial pathway [162]. Meng et al. designed UA derivatives through multiple synthetic steps and evaluated the synthesized compounds in vitro using an MTT assay on two cancer cell lines (BEL7402 and SGC7901). The results showed a higher inhibitory rate of the synthesized compounds on both cells when compared to the parent compound, UA. The results of molecular docking studies indicated the role of the structural design and optimization of UA. The most promising derivative was compound **24,** as shown in Table 4 [163].

Wolfram et al. converted pentacyclic triterpenoic acids—namely oleanolic, betulinic glycyrrhetinic, boswellic, and ursolic acids—into their acetylated piperazinyl amides by coupling them with rhodamine B. To evaluate their in vitro cytotoxicity, all the triterpene-homopiperazinyl-rhodamine derivatives were subjected to RB assays and most of them were highly toxic against numerous human cancer cell lines (A2780, A375, HT29, NiH3T3, MCF7, and SW1736). The ursolic acid-homopiperazinyl-rhodamine derivative (Compound **25**, Table 4) was one of the compounds that showed a strong cytotoxicity against the tumor cell lines, while it was less cytotoxic on SW1736 cells [164]. Kahnt et al. synthesized amine-spaced conjugates of UA and 1,4,7,10-tetraazacyclododecane-1,4,7,10-tetraacetic acid (DOTA). Two conjugates (Compound **26**, **27**, Table 4) displayed the highest cytotoxicity on A375 melanoma and A2780 ovarian carcinoma, with IC_50_ values of 1.5 µM and 1.7 µM, respectively. Compound **26** induced the death of A375 cells by apoptosis [165]. Mang et al. synthesized UA derivatives with potential anticancer properties by modifying C-2, C-3, and C-28 and evaluated their in vitro cytotoxicity against HepG2, BGC-823, and HeLa cell lines using an MTT assay These derivatives were synthesized by reacting UA with Jones reagents in acetone and then with NH_2_-OH·HCl; the obtained compounds were then reacted with Ac_2_O in the presence of DMAP in THF. The intermediate compound was condensed with the relevant amino or phenolic moieties in Et_3_N to afford the targeted compounds. Among these derivatives, Compounds **28** and **29** were demonstrated to be more effective on the cell lines when compared to the reference drug, gefitinib [166].

#### 3.1.3. Modification of β-Hydroxy (C-3 Position)

Fontana et al. synthesized derivatives of UA and oleanolic acid with a modified oxidation state and lipophilicity at C-3 and C-28 positions which were screened in vitro on hepatocarcinoma cell lines—namely HepG2, HA22T/VGH, and Hep3B. UA derivatives containing three carbons as the side chain at the C-3 position of UA were synthesized in stereoisomeric forms using the Barbier–Grignard method and (Compounds **30** and **31**, Table 5) were found to be effective against all three cancer cell lines; these compounds inhibited cell growth and induced the inhibition of NF-κB activation in the cell lines [167].

**Figure 3:**
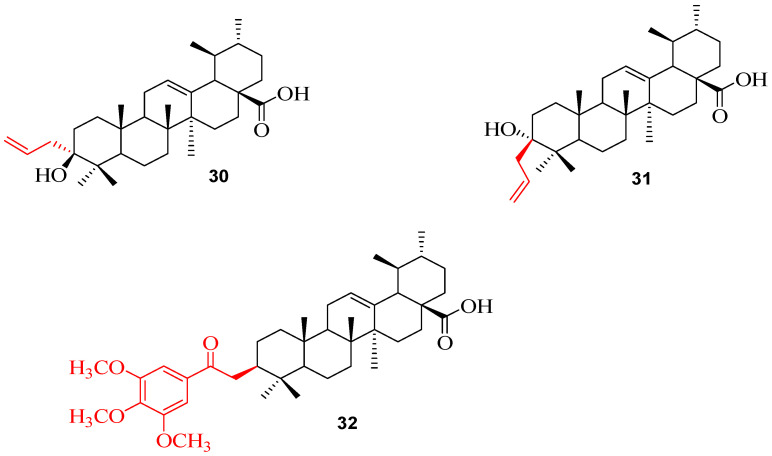
Fontana and Xu ‘s UA derivatives.

Xu et al. isolated triterpenoids from the acorn-starch/licorice and reacted them with 3,4,5-methoxybenzoic acid under dicyclohexyl carbodiimide (DCC)/DMAP conditions and evaluated their cytotoxicity in four cells, including A-549, MCF-7, H1975, and BGC-823. Most of the synthesized compounds indicated a significant cytotoxic activity in all the four cell lines and a lower toxicity in the normal cells, Human Hair Dermal Papilla Cells (HHDPC) when compared to the positive control, mitomycin C. The UA derivative containing 3,4,5-methoxy-phenacyl at the C-3 position (compound **32**, Figure 3, Table 5) was the derivative with a significant antiproliferative effect, with IC_50_ values in the range of 6.07–22.27 µM [140].

#### 3.1.4. Modification of Miscellaneous Groups

**Scheme 4:**
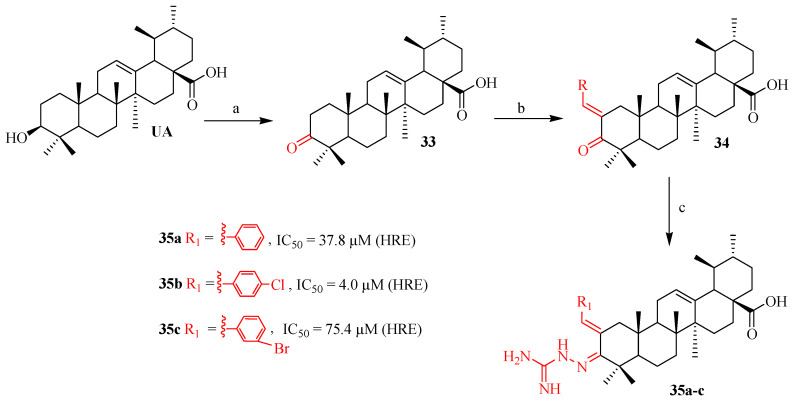
Synthesis of compounds **33**–**35**. Reagents and conditions: (**a**) Jones reagent acetone, 0 °C, 5 h, 90%; (**b**) Aldehydes, 5% NaOH, absolute EtOH, r.t 2 h, 30–75%; (**c**) 37% HCl, absolute EtOH, reflux, 8 h, 34–65%.

Wu et al. synthesized UA derivatives bearing an aminoguanidine moiety and investigated them as HIF-1α inhibitors and anticancer agents in human cancer cell lines. The majority of these compounds showed a potent inhibition of the HIF-1α transcriptional effect; among these derivatives, compound **35b** (Scheme 4) was found to be the most potent inhibitor of HIF-1α expression in hypoxic conditions (IC_50_ 4.0 µM), with no significant cytotoxicity noted against any cell lines tested [168].

**Scheme 5:**
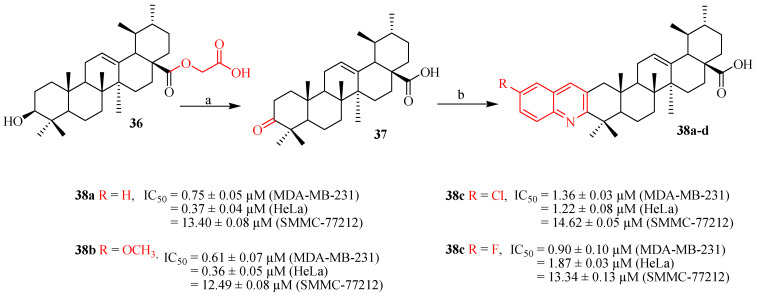
Synthesis of compounds **36**–**38**. Reagents and conditions: (**a**) Jones reagent acetone, 0 °C, 5 h; (**b**) EtOH, substituted o-amino benzaldehyde, KOH, reflux under N_2_ atmosphere for 24 h.

Gu et al. designed and synthesized a series of new ursolic acid-based derivatives through the conjugation of UA with quinolone and oxadiazole motifs and investigated their anticancer activity. These compounds were investigated in vitro on human cancer cell lines-namely MDA-MB-231, Hela, and SMMC-7721, and the results indicated that compounds **36**–**38** exhibited a strong growth inhibitory effect against the three cancer cell lines. Among these derivatives, compound **38b** exhibited the most potent antitumor activity, with IC_50_ values of 0.61 ± 0.07 (MDA-MB-231), 0.36 ± 0.05 (HeLa), and 12.49 ± 0.08 µM (SMMC-7721) when compared to the positive control, etoposide [169] (Scheme 5).

**Scheme 6:**
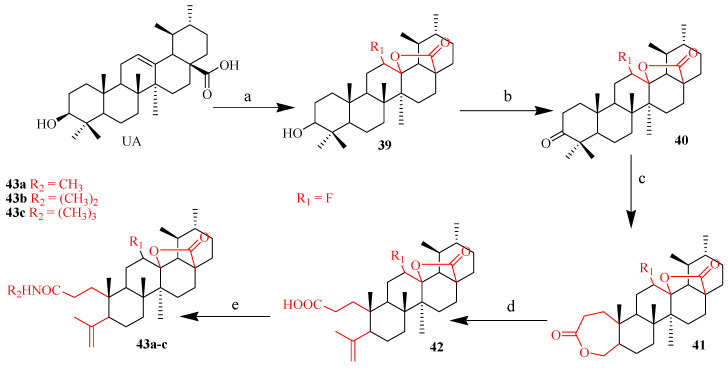
Reagents and conditions: (**a**) Selectfluor®, dioxane, nitromethane, 80 °C, 24 h; (**b**) Jones reagent, acetone, ice; (c) m-CPBA 77%, CHCl_3_, r.t., 120 h; (d) p-toluenesulfonic acid monohydrate, CH_2_Cl_2_, r.t., 24 h; (e) R_2_NH_2_, dry THF, Et_3_N, T3P (50 wt% in THF), ice.

Mendes et al. synthesized ring-A-cleaved UA derivatives and evaluated their antiproliferative activity against three lung cancer cell lines—namely H460, H322, and H460^+/+^—using 2D or 3D culture models. Three of these newly designed UA derivatives bearing a cleaved ring-A with a secondary amide at C-3 (compounds **43a**–**c**, Scheme 6) demonstrated significantly enhanced antiproliferative effects in the 2D systems. These compounds possessed a potent anticancer activity and the preliminary mechanism of action showed that compound **43c** induced apoptosis through the activation of caspase-7 and caspase-8 and the decrease in Bcl-2 [170].

**Scheme 7:**
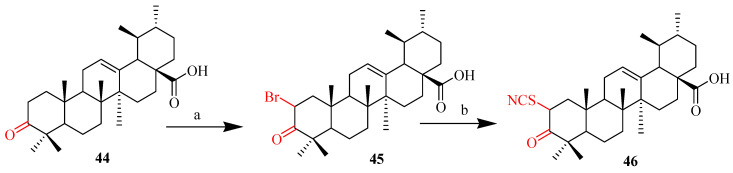
Reagents and conditions: (**a**) CuBr_2_, EtOAc, MeOH, r.t., 3 h; (**b**) KSCN, DMSO, 90 °C, 24 h.

Borkoa et al. synthesized the novel triterpenic derivatives of dihydrobetulonic, betulonic, and ursonic acid. All of these compounds were tested for their in vitro cytotoxic activity against human cancer cell lines—namely CCRF-CEM, CEM-DNR, HCT116, HCT116 p53^−/−^, K562, K562-TAX, A549, U2OS, and two noncancerous fibroblasts such as BJ and MRC-5. Compounds **45** and **46** were the most effective on the CCRF-CEM cell lines and less toxic in non-cancerous fibroblasts. These derivatives triggered apoptosis via the intrinsic pathways. The ursonic acid derivative **45** was synthesized by the bromination of UA using copper (II) bromide in a solution of ethyl acetate (EtOAc) and methanol (MeOH) at room temperature. Compound **46** was obtained by the nucleophilic substitution of bromoketone **43** by potassium thiocyanate (KSCN) in DMSO at 90 °C, as shown in Scheme 7 [171].

**Scheme 8:**
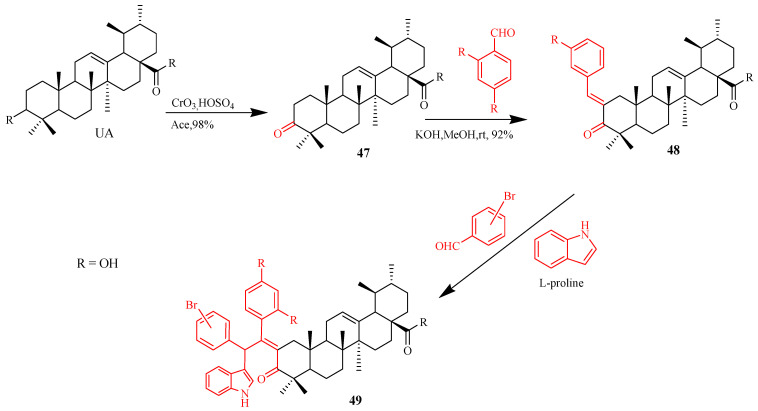
Synthesis of UA hybrid compounds.

Fan and colleagues synthesized UA hybrid compound **49** (Scheme 8) by reacting UA with Jones reagent at 0 °C in dimethyl ketone to obtain a C-3 oxidized derivative. The oxidized compound was then reacted with benzaldehyde using ethanolic potassium hydroxide (KOH) via the Claisen Schmidt condensation reaction at room temperature to achieve a benzylidine hybrid compound. The benzylidine compound was then reacted with an indole and substituted with a benzaldehyde in EtOAc at room temperature for 2 h to achieve compound **49**. The anticancer potential of the compound against glioma cells was studied. The compound demonstrated a good inhibition of cell proliferation and induced apoptosis when compared to the parent compound, UA [172].

**Figure 4:**
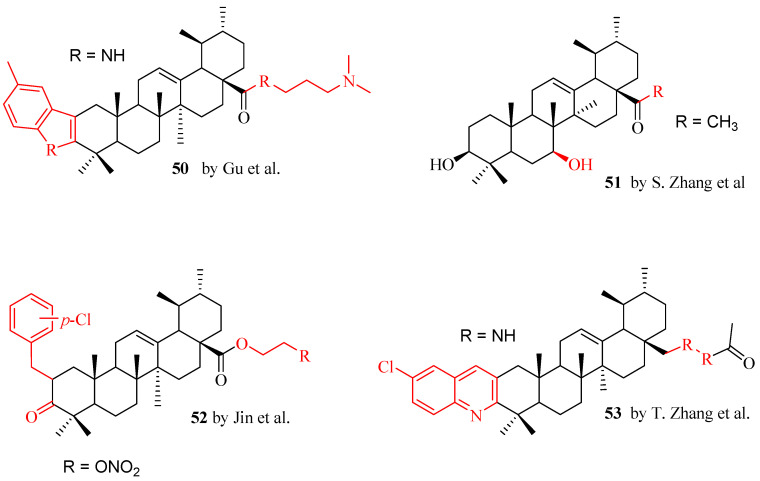
Gu’s, S. Zhang’s, Jin’s and T. Zhang’s work on UA derivatives.

An aromatic heterocyclic compound containing carbazole has attracted attention due its potential anticancer activity [173]. Gu et al. synthesized a series of carbazole derivatives of UA. Among these derivatives, compound **50** (Figure 4) showed a significant cytotoxic activity against the hepatocarcinoma cell line, HepG2, with an IC_50_ value of 1.26 ± 0.17 µM [174]. Zhang et al. employed the biotransformation of UA by *Mucor spinosus* AS 3.3450, and three novel compounds were isolated. Compound **51** (Figure 4), identified as 3β, 7β-dihydroxy-ursolic acid-28-etha-none indicated a stronger cytotoxic activity against the tumor cell lines (Hela, K562, and KB) when compared to the parent compound UA [175]. A number of NO-donating ursolic acid-based benzylidene derivatives with various substitutions were synthesized by Zhang et al. They were further analyzed for their in vitro cytotoxicity against the HepG-2, MCF-7, HT-29, and A549 cancer cell lines. Most of these derivatives revealed a weaker inhibitory effect when compared to UA; compound **52** (Figure 4) showed the most potent activity against HT-29 (IC_50_ = 4.28 μM). The further investigation of its anticancer mode of action revealed that it induced the apoptosis of HT-29 cell lines in a dose-dependent manner and indicated cell cycle arrest at the G1 phase, which led to cell apoptosis and also induced apoptosis via the mitochondria-mediated pathways [176].

Jin et al. synthesized UA-based quinoline derivatives bearing thiadiazole, hydrazide, or oxadiazole moieties. The in vitro antiproliferative activity on tumor cells such as SMMC-7721, MDA-MB-231, and HeLa revealed that quinoline-based derivatives bearing carboxyl moieties or hydrazide moieties exhibited a significant anticancer activity against all the cancer cell lines when compared to the positive control, etoposide. Furthermore, a pharmacological in vitro analysis revealed that compound **53** (Figure 4) exhibited an antiproliferative activity against HeLa cell lines by cell cycle arrest at the G0/G1 phase, decreasing the mitochondrial membrane potential, inducing intracellular ROS generation, intervening with the Ras/Raf/mitogen-activated protein kinase kinase (MEK)/extracellular signal-regulated kinase (ERK) signaling pathways as MEK kinase inhibitors, and finally inducing the apoptosis of HeLa cell lines. The molecular docking analysis also revealed compound **53** capability to effectively bind with the active site of MEK [177]. 

The 1C_50_ values of compound 35b, 38b, 43a–b, 45, 46 and 50–53 on different cancer cell lines are shown in Table 6.

## 4. Insights and Future Directions

This review reports various strategies that have been used to design UA-based derivatives with enhanced anticancer activity when compared to UA or conventional drugs used as controls in most of the studies reported over the past five years. Many studies on pentacyclic triterpenoids have shown that the C-2 position, β-hydroxyl (C-3), and carboxylic moieties (C-28) of UA were the major sites for the modification of its structure. Most researchers modified the molecular structure of UA around the three sites, resulting in the improvement in the chemical or physical activities of the UA molecule.

The derivatives were grouped according to their active sites of modifications. Their corresponding IC_50_ values are listed in the tables against reference compounds, usually UA or a conventional drug. The derivatives were tested against various cancer cell lines in vitro using different cell lines of colon, prostate, gastric, leukaemia, lung, breast, pancreas, skin, glioblastoma, and renal cells. Most of the derivatives demonstrated an improved antiproliferative activity when compared to their respective reference molecules, revealing that UA structural modification significantly enhanced their antiproliferative activity.

Despite the great progress which has been made, there are still a lot of research gaps, such as the synthesis of ursolic acid-based hybrid compounds via hybridization with known chemotherapeutic scaffolds, bioavailability studies, and toxicological studies. Furthermore, more evaluation in vivo is lacking and there is a pressing need to evaluate the synthesized compounds in vivo.

## Figures and Tables

**Table 1:** Several plant species with (ursolic acid) UA constituent.

Plant Species (Family)	Plant Parts Used	Bioactivities	Bibliography
*Arctostaphylos uva-ursi* (L.) Spreng (Ericaceae)	Leaves	Antitumor, antibacterial	[15,16]
*Argania spinosa* (L.) Skeels (Sapotaceae)	Fruits, leaves	Antibacterial, antifungal	[17,18]
*Bouvardia ternifolia* (Cav.) Schltdl. (Rubiaceae)	Aerial parts	Anti-Alzheimer	[19]
*Bursera cuneata* (Schldl.) Engl (Burseraceae)	Aerial parts (stems and leaves)	Anti-inflammatory, antihistaminic	[20]
*Catharanthus roseus* (L.) G. Don (Apocynaceae)	Leaves	Anticancer	[21]
*Cornus mas* (L.) (Cornaceae)	Fruits	Antitumor	[22]
*Eriobotrya japonica* (Thunb.) Lindl (Rosaceae)	Leaves	Anti-cancer, anti-osteoclastic, skin disorder anti-inflammatory, and anti-arthritic	[23,24,25,26,27]
*Eucalyptus globulus *(Labill.) (Myrtaceae)	Leaves, bark	Antioxidant, neuroprotective	[28,29]
*Fragraefragrans* (Roxb.) (Gentianaceae)	Leaves, fruits, bark	Antimycobacterial	[30]
*Ilex aquifolium* (L.) (Aquifoliaceae)	Leaves	Anticancer, antimalarial, antibacterial	[31]
*Lamium album* (L.) (Lamiaceae)	Flowers	Antioxidant and anti-inflammatory	[32]
*Lantana Camara* (L.) (Verbenaceae)	Leaves	Antifungal, antiproliferative, anti-diabetes, anxiolytic	[33,34,35]
*Lepidozia chordulifera* (Dumort.) (Porellaceae)	Leaves	Antibacterial	[36]
*Ligustrum lucidum* (Ait.) (Oleaceae)	Fruits	Coronary heart disease and diabetes	[37,38]
*Malus domestica* (sp.) (Rosaceae)	Fruits, leaves	Antioxidant	[39]
*Malus pumila* (Mill.) (Rosaceae)	fruits	Antitumor	[40]
*Ocimum forskolei* (Benth.) (Lamiaceae)	Aerial parts (leaves and stems)	Antiulcer	[41]
*Ocimum sanctum* (L.) (Lamiaceae)	leaves	Induced arthritis, antiproliferative, anti-stress	[42,43,44]
*Panax ginseng* (C.A. Mey.) Baill. (Araliaceae)	Roots and rhizomes	Anticancer, antiviral	[45]
*Paulownia tomentosa* (Thunb.) Steud. (Scrophulariaceae)	Leaves	Anticancer	[46]
*Prunella vulgaris* (L.) (Lamiaceae)	Aerial parts	Antiviral, antiestrogenic	[47,48]
*Psidium guajava* (L.) (Myrtaceae)	Leaves	Hypoglycaemic, antimicrobial	[49]
*Rabdosia rubescens* (Linn.) (Lamiaceae)	Anti-tumour	Antitumor	[50]
*Rosmarinus officinalis* (L.) (Lamiaceae)	Stems and leaves	Antidepressant	[51]
*Sambucus australis* (Cham. & Schltdl.) (Adoxaceae)	Aerial parts	Antibacterial and Antioxidant	[52]
*Saurauja roxburghii* (Wall.) (Dilleniaceae)	Leaves	Anticancer	[53]
*Thymus vulgaris* (L.) (Lamiaceae)	Aerial parts (stems and leaves)	Anticancer, cardiovascular, antihyperlipidemic, antioxidant, antifungal	[54,55,56]
*Tribulus arabicus* (Hosni.) (Zygophyllaceae)	Aerial parts	Antihyperuricemic, antioxidant	[57,58]
*Paulownia tomentos* (Thunb.) Steud. (Scrophulariaceae)	Fruits	Anticancer	[46]
*Punica granatum* (Linn.) (Punicaceae)	Flowers	Antioxidant, antidiabetic	[59,60]
*Uncaria rhynchophylla* (Gouteng.) (Rubiaceae)	Stems and hooks	Anticancer	[61,62]
*Vitex negundo* (L.) (Lamiaceae)	Leaves	Antibacterial, antifeedant against the larvae	[63]
*Ziziphus jujuba* (Mill.) (Rhamnaceae)	Leaves	Anticancer, anti-obesity, and antioxidant	[64]

**Table 2:** Reports of ursolic acid’s significant pharmacological activities.

Sr. No.	Pharmacological Activities	Bibliography
1	Antioxidant	[102,103,104]
2	Antibacterial	[52,67,105,106,107,108,109,110]
3	Antifungal	[111,112,113,114,115,116,117]
4	Anticancer	[13,118,119,120,121,122,123]
5	Antidiabetic	[34,124,125,126,127,128]
6	Anti-inflammatory	[129,130,131,132,133]
7	Antiviral	[134,135,136]

**Table 3:** IC_50_ values of UA derivatives modified on the carboxylic moiety (C-28).

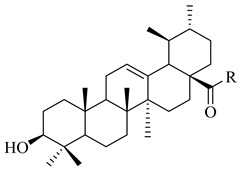					
Compound	R	Biological Activity	Cell Lines Tested IC_50_ (µM)	Reference Molecules IC_50_ (µM)	Bibliography
**9a**	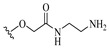	Antiproliferative	MCF-7 (8.45 ± 0.26) Hela (8.37 ± 0.11) A549 (10.06 ± 1.39)	MCF-7 (Gefitinib) 17.83 ± 7.85 Hela (Gefitinib) 15.40 ± 4.65 A549 (Gefitinib) 11.02 ± 3.27	[155]
**13b**	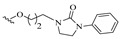	Anticancer activity	HRE (36.9)	n.d	[156]
**14**	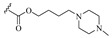	Anticancer activity	MGC-803 (4.99 ± 0.40) Bcap-37 (8.56 ± 0.44)	MGC-803(UA) 26.51 ± 1.1 Bcap-37(UA) 31.39 ± 0.85	[157]

**Table 4:** Modification of C-3 and C-28 positions.

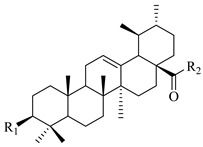						
Compound	R_1_	R_2_	Biological Activities	Cell Lines Tested(IC_50_µM)	Reference Molecules (IC_50_µM)	Bibliography
**15**	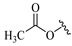	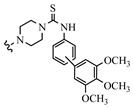	Cytotoxicity	MGC-803 (9.82 ± 0.29) HCT-116 (18.97 ± 0.53) T24 (19.60 ± 0.43) HepG2 (15.72 ± 0.84) A549 (20.79 ± 0.54) HL-7702 (˃100)	MGC-803(UA) 27.08 ± 0.29 HCT-116(UA) 38.78 ± 0.16 T24(UA) 29.29 ± 0.80 HepG2(UA) 30.21± 0.58 A549(UA) 35.79 ± 0.37 HL-7702(˃100)	[139]
**16**	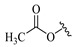	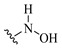	Cytotoxicity	518A2 (3.6 ± 0.1) A2780 (2.7 ± 0.1) A549 (3.9 ± 0.1) FaDu (6.4 ± 0.4) HT29 (3.5 ± 0.3) MCF-7 (3.3 ± 0.2) NIH 3T3 (2.5 ± 0.6)	518A2 (UA) 14.7 ± 0.1 A2780 (UA) 11.7 ± 0.6 A549 (UA) 15.5 ± 1.3 FaDu (UA) 14.2 ± 2.0 HT29 (UA) 10.6 ± 0.3 MCF-7 (UA) 12.7 ± 0.1 NIH 3T3 (UA)18.7 ± 1.6	[158]
**17**	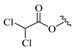	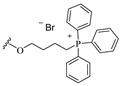	Cytotoxicity	TET21N (0.81 ± 0.08) MCF-7(1.59 ± 0.11)	TET21N (˃10) MCF-7(˃25)	[159]
**18**	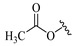	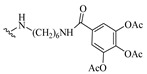		T24(6.01 ± 0.87) A549(5.22 ± 0.65) HepG2(6.82 ± 1.07) SKOV3(8.95± 1.26)	T24(UA)37.88 ± 1.12 A549(UA) HepG2(UA) SKOV3(UA)	[94]
**19**	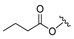	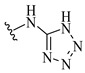	Anti-cancer	HRE(0.8 ± 0.2)	HRE (UA) > 100	[160]
**20**	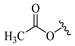	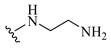	Cytotoxicity	518A2(2.7 ± 0.10) A2780(2.3 ± 0.10) HT29(1.8 ± 0.10) MCF-7(2.0 ± 0.10) 8505C(4.1 ± 0.40) NIH3T3(2.6 ± 0.30)	518A2(UA) 14.7 ± 0.1 A2780(UA) 11.7 ± 0.6 HT29(UA) 10.6 ± 0.7 MCF-7(UA) 12.7 ± 0.1 8505C(UA) 13.5 ± 1.5 NIH 3T3(UA) 18.7 ± 1.6	[161]
**21**	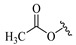	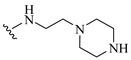		518A2(3.2 ± 0.10) A2780(2.4 ±0.10) HT29(1.8 ± 0.20) MCF-7(2.7 ± 0.30) 8505C(5.4 ± 0.40) NIH 3T3(2.2 ± 0.10)	518A2(UA) 14.7 ± 0.1 A2780(UA) 11.7 ± 0.6 HT29(UA) 10.6 ± 0.7 MCF-7(UA) 12.7 ± 0.1 8505C(UA) 13.5 ± 1.5 NIH 3T3(UA) 18.7 ± 1.6	
**22**	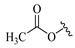	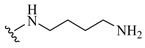		518A2(2.7 ± 0.10) A2780(2.6 ± 0.10) HT29(1.7 ± 0.10) MCF-7(1.7 ± 0.10) 8505C(3.2 ± 0.01) NIH 3T3 (1.3 ± 0.20)	518A2(UA) 14.7 ± 0.1 A2780(UA) 11.7 ± 0.6 HT29(UA) 10.6 ± 0.7 MCF-7(UA) 12.7 ± 0.1 8505C(UA) 13.5 ± 1.5 NIH 3T3(UA) 18.7 ± 1.6	
**23**	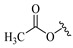	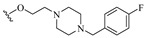	Cytotoxicity	Hela (2.6 ± 1.1) MKN45(2.1 ± 0.3)	Hela (Cisplatin) 15.1 ± 0.9 MKN45(Cisplatin) 2.8 ± 0.1	[162]
**24**	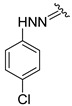	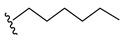	Cytotoxicity	BEL-7402 (4.49) SGC-7901(7.01)	BEL-7402 (UA) >50 SGC-7901(UA) >50	[163]
**25**	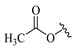	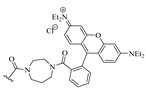	Cytotoxicity	A375(0.51 ± 0.05) A2780(0.45 ± 0.03) HT29(0.50 ± 0.07) MCF7(0.39 ± 0.04) NiH3T3(0.40 ± 0.03) SW1736 (n.d)	A375(BA) A2780(BA) HT29(BA) MCF7(BA) NiH3T3(BA) SW1736(BA)	[164]
**26**	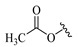	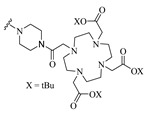	Cytotoxicity	A375(1.5 ± 0.4) A2780(1.9 ± 0.3) HT29 (5.7 ± 0.5) MCF-7(4.4 ± 0.7) FaDu (3.7 ± 0.6) NIH 3T3(4.6 ± 1.0)	A375(UA) n.d. A2780(UA) 11.7 ± 0.6 HT29 (UA) 10.6 ± 0.7 MCF-7(UA) 12.7 ± 0.1 FaDu (UA) n.d NIH 3T3(UA) 13.1 ± 1.1	[165]
**27**	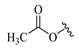	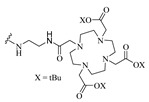		A375(2.0 ± 0.1) A2780(1.7 ± 0.1) HT29 (2.3 ± 0.3) MCF-7(1.8 ± 0.1) FaDu (2.0 ± 0.2) NIH 3T3(1.4 ± 0.1)	A375(UA) n.d. A2780(UA) 11.7 ± 0.6 HT29 (UA) 10.6 ± 0.7 MCF-7(UA) 12.7 ± 0.1 FaDu (UA) n.d NIH 3T3(UA) 13.1 ± 1.1	
**28**	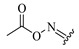	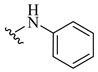	cytotoxicity	HeLa(9.25) HepG2(21.2) BGC-823(8.06)	HeLa(Gefitinib) 17.1 HepG2(Gefitinib) 20.7 BGC-823 Gefitinib 19.3	[166]
**29**	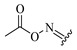	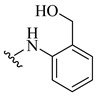		HeLa(13.8) HepG2(23.7) BGC-823(9.15)		

n.d = not determined. BA = Betulinic acid.

**Table 5:** Modification of β-hydroxy (C-3).

Compound	Biological Activity	Cell Lines Tested (IC_50_µM)	Reference Molecules (IC_50_µM)	Bibliography
**30**	Cytotoxicity	HA22T/VGH(31.0 ± 1.5) HepG2(28.0 ± 2.0) Hep3B(32.5 ± 2.5)	HA22T/VGH(UA) ˃ 100 HepG2(UA) ˃ 100 Hep3B(UA) ˃ 100	[167]
**31**	Cytotoxicity	HA22T/VGH(31.0 ±1.5) HepG2(28.0 ± 2.0) Hep3B(32.5 ± 2.5)	HA22T/VGH(UA) ˃100 HepG2(UA) ˃ 100 Hep3B(UA) ˃ 100	[167]
**32**	Cytotoxicity	A549 (6.07 ± 0.91) H1975(10.64 ± 1.94) MCF-7(22.27± 3.51) BGC-823 (17.10 ± 1.04) t-HSC/Cl-6 (29.12 ± 3.71)	A549 (mitomycin C) 28.14 ± 3.41 H1975(mitomycin C) 34.51 ± 3.06 MCF-7(mitomycin C) 44.08 ± 4.01 BGC-823 (mitomycin C) 37.94 ± 2.88 t-HSC/Cl-6 (mitomycin C) n.d	[140]

n.d = not determined.

**Table 6:** Modification of miscellaneous groups.

Compound	Activity	Cell Lines Tested (IC_50_µM)	Reference Molecules (IC_50_µM)	Bibliography
**35b**	Cytotoxicity	HRE (4.0)	HRE(UA) >100	[168]
**38b**	Cytotoxicity	MDA-MB-231 (0.61 ± 0.07) HeLa (0.36 ± 0.05) SMMC-7721(12.49± 0.08) QSG-7701( > 40)	MDA-MB-231(UA) > 40 HeLa (UA) >40 SMMC-7721(UA) >40 QSG-7701(UA) n.d	[169]
**43a**	Cytotoxicity	H460(4.5 ± 0.4) H322(6.8 ± 1.5) H460(6.7 ± 0.5)	H460(UA) 14.8 ± 0.6 H322(UA) 15.3 ± 2.8 H460 LKB1^+/+^(UA) 21.1 ± 1.6	[170]
**43b**		H460(5.3 ± 0.3) H322(7.3 ± 1.0) H460 LKB1^+/+^(7.8 ± 1.1)		
**43c**		H460(2.6 ± 0.9) H322(3.3 ± 0.9) H460 LKB1^+/+^(4.4 ± 0.6)		
**45**	Cytotoxicity	CCRF-CEM (3.6) CEM-DNR (21.8) HCT116 (28.4) HCT116 p53^−/−^(29.8) K562 (38.8) K562-TAX (25.1) A549 (27.6) U2OS (20.7) BJ (49.5) MRC-5(29.3)	CCRF-CEM14 (**44**) 10.4 CEM-DNR (**44**) 34.0 HCT116(**44**) 34.0 HCT116 p53^−/−^(**44**) 49.7 K562 (**44**) >50 K562-TAX (**44**) 35.5 A549 (**44**) >50 U2OS (**44**) >50 BJ (**44**) >50 MRC-5(**44**) >50	[171]
**46**		CCRF-CEM (4.7) CEM-DNR (28.2) HCT116 (32.1) HCT116 p53^−/−^(32.3) K562 (34.7) K562-TAX (29.0) A549 (42.5) U2OS (33.1) BJ (˃ 50) MRC-5(39.8)		
**50**	Cytotoxicity	SMMC-7721 (1.08 ± 0.22) HepG2 (1.26 ± 0.17)	SMMC-7721(Doxorubicin) 0.62 ± 0.16 HepG2 (Doxorubicin) 0.77 ± 0.12)	[174]
**51**	Cytotoxicity	Hela (1.06) K562 (28.7) KB(35.6)	Hela (UA) 14.2 K562 (UA) 52.7 KB 3(UA) 42.9	[175]
**52**	Cytotoxicity	HepG-2(65.8 ± 6.3) MCF-7(> 100) HT-29(4.28 ± 3.5) A549(78.39 ± 5.6)	HepG-2 (UA) 44.35 ± 4.9 MCF-7 (UA) > 100 HT-29 (UA) > 100 A549 (UA) > 100	[176]
**53**	MEK inhibitors	MDA-MB-231 (1.84 ± 0.13) HeLa (1.18 ± 0.03) SMMC-7721 (17.48 ± 0.10) QSG-7701(40.59 ± 2.89)	MDA-MB-231 (Etoposide) 5.26 ± 1.21 HeLa (Etoposide) 2.98 ± 0.42 SMMC-7721 (Etoposide) 3.48 ± 0.35 QSG-7701(Etoposide) 28.75 ± 3.28	[177]

n.d = not determined.

## Abbreviations

FDA	Food and Drug Administration
UA	Ursolic acid
P53	Tumor protein p53
Wnt	Wnt/β-catenin pathways
Ras	Retrovirus-associated DNA sequences
TRAIL	TNF-related apoptosis-inducing ligand
STAT3	Signal transducers and activators of transcription
PK	Pharmacokinetic
UV	Ultraviolet
HIF-1α	Hypoxia-inducible factor 1-alpha
DMF	Dimethylformamide
MTT	Dye compound 3-(4,5-Dimethylthiazol-2-yl)-2,5-diphenyltetrazolium bromide assay
SRB	sulforhodamine B assay
PARP-1	Poly [ADP-ribose] polymerase 1
NF-kB	Nuclear factor kappa-B
DMAP	4-Dimethylaminopyridine
DCM	Dichloromethane
THF	Tetrahydrofuran
DCC	*N*,*N*′-Dicyclohexylcarbodiimide
DMSO	Dimethyl sulfoxide
FROS	Reactive oxygen species
MEK	Mitogen-activated extracellular signal-regulated kinase
